# Typhoid fever cases in the U.S. military

**DOI:** 10.1186/s12879-015-1159-6

**Published:** 2015-10-14

**Authors:** Tia Sorrell, Daniel J. Selig, Mark S. Riddle, Chad K. Porter

**Affiliations:** Enteric Diseases Department, Infectious Disease Directorate, Naval Medical Research Center, 503 Robert Grant Avenue, Silver Spring, MD 20910 USA

**Keywords:** Salmonella typhi, Travel medicine, Military health

## Abstract

**Background:**

*Salmonella enterica*, serovar Typhi (S. Typhi), a causative agent of enteric fever (typhoid fever), predominately affects populations in developing regions with poor access to clean food and water. In addition, travelers to these regions are at risk of exposure.

**Methods:**

We report the epidemiological characteristics of S. Typhi cases among active duty United States military personnel from 1998 to 2011 using data obtained from the Defense Medical Surveillance System. Cases were identified based on International Classification for Disease Ninth Edition - Clinical Modification codes.

**Results:**

We identified a total of 205 cases S. Typhi for an incidence of 1.09 per 100,000 person-years. Cases were on average 31.7 years old, predominately married (*n* = 129, 62.9 %), Caucasian (*n* = 142, 69.3 %), male (*n* = 176, 85.9 %), and had a high school education (*n* = 101, 49.3 %). Of the identified cases, 122 had received a Typhoid vaccination within 4 years of diagnosis.

**Conclusion:**

This study provides an overview of enteric fever in the United States military. The incidence was similar to the general U.S. population except for increased incidence from 1998 to 2000, perhaps attributable to operational deployments in that period. Given that vaccination is an effective primary prevention measure against typhoid fever, active monitoring of pre-deployment vaccine history is warranted.

## Background

Typhoid fever, caused by infection with the *Salmonella enterica*, serovar Typhi (S. Typhi), is transmitted by ingestion of food or beverage contaminated with fecal matter, and accounts for a considerable burden of disease in areas lacking clean water or satisfactory sanitation [1, 2]. Over 21 million cases of typhoid fever and 200,00 attributable deaths occur globally each year with high incidence estimates of greater than 100 cases per 100,000 person-years (100 K p-y) in south-central and south-east Asia [3]. However, in communities that have access to clean water and adequate sanitation such as Europe, Australia and North America, typhoid fever is relatively uncommon with low incidence estimates of less than ten cases per 100 K p-y. These cases are usually related to travel to endemic areas to include travelers visiting friends and relatives [4, 5].

Essential measures to prevent infection with S. Typhi are safe water access, safe food handling practices, sanitation measures, and public education. Vaccination is also an important precaution to prevent enteric infection, especially in individuals travelling to endemic areas, during outbreak, or routinely in school-aged children wherever the control of disease is a priority [2, 6]. Currently, two typhoid vaccines are commercially available in the U.S., the Ty21a (oral) vaccine and the Vi polysaccharide (parenteral) vaccine [7]. In the past, two additional parenteral typhoid vaccines were also commonly administered, an acetone-inactivated (AKD) parenteral vaccine (only available to the U.S. armed forces) and a heat-phenol (H-P) inactivated vaccine. However, the use of AKD and H-P inactivated vaccines has become obsolete secondary to poor side-effect profiles and because these vaccines are not considered to be more effective than other available typhoid vaccines (Table 1) [6, 8–10].

**Table 1:** Summary of typhoid fever vaccines [6, 13–16]

Typhoid vaccine	Route	3-year efficacy	Booster	Adverse reactions	
				Local	Systemic
Vi Polysaccharide	Parenteral	30–70 %	2 years	10–40 %	2 %
Live Ty21a	Oral	34–58 %	5 years	NA	<1 %
Parenteral heat-phenol inactivated	Parenteral	51–77 %	3 years	10–50 %	10–20 %
Acetone-inactivated	Parenteral	75–94 %	Not applicable	10–50 %	10–20 %

We reviewed data of cases of typhoid fever from the Defense Medical Surveillance System, identified based on an International Classification for Disease Ninth Edition - Clinical Modification from 1998–2011. Our primary goal was to describe the epidemiology of S. Typhi cases in the U.S. military.

## Methods

S. Typhi cases were identified from all active duty U.S. military personnel from 1998 to 2011. Cases were defined based on a medical encounter in which the International Classification of Disease, Ninth Revision-Clinical Modification (ICD-9-CM) code for Typhoid fever (002.0) was assigned. Descriptive analyses included estimates of S. Typhi vaccination within 4 years of infection and the frequency of documented cases among U.S. military populations over time and across multiple demographic characteristics. All statistical analyses were performed using SAS v. 9.2 for Windows (SAS Institute, Cary, NC).

Medical encounter data were obtained from the Defense Medical Surveillance System (DMSS) which includes Standard Ambulatory Data Record (SADR), Comprehensive Ambulatory Patient Encounter Record (CAPER) and Standard Inpatient Data Record (SIDR) claims data for care obtained within the Military Health Services and the Tri-Service Reportable Events System data as well as demographic information from personnel data and immunization data from the Defense Enrollment Eligibility Reporting System (DEERS). Data were supplied by the Armed Forces Health Surveillance Center (AFHSC), which oversees the DMSS [11]. Military population demographic data were provided by the Defense Medical Data CenterPrior to providing the data to investigators, personnel at AFHSC linked all available information on selected subjects using the defined ICD-9 codes and requested fields into SAS data files and removed all individually identifiable information.

The study protocol was approved by the Naval Medical Research Center Institutional Review Board in compliance with all applicable Federal regulations governing the protection of human subjects.

## Results

**Fig. 1:**
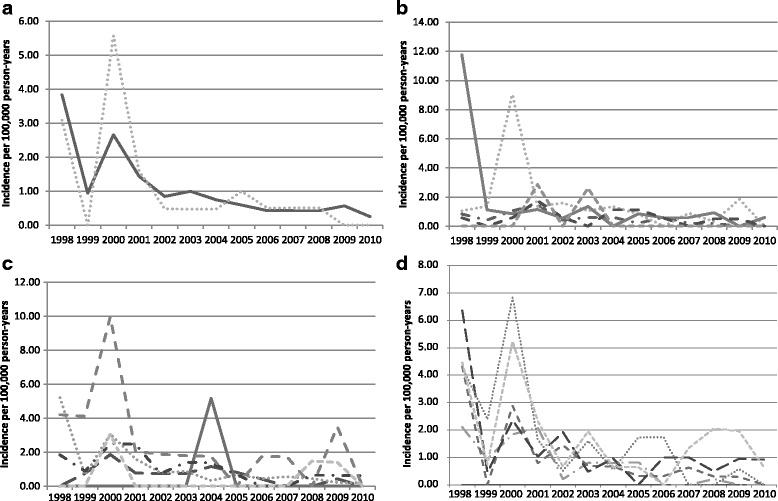
**a**. S. Typhi incidence by gender . **b**. S. Typhi incidence by service . **c**. S. Typhi incidence by race . **d**. S. Typhi incidence by age

Between 1998 and 2011, 205 cases of typhoid fever were identified among U.S. military service members (Table 2). The mean age of identified cases was 31.7 (standard deviation: 7.6) years and cases were predominately (*n* = 129, 62.9 %), Caucasian (*n* = 142, 69.3 %), male (*n* = 176, 85.9 %), and had a high school education (*n* = 101, 49.3 %). Furthermore, subjects were more commonly Navy (*n* = 78, 38.1 %) or Air Force (*n* = 73, 35.6 %) service members (Fig. 1a–d). Prior to 2000, S. Typhi incidence among males was non-significantly higher than that of their female counterparts with 2.41 cases per 100 K p-y as opposed to 1.55 cases per 100 K p-y in females (*p* = 0.30). Notably, there was a marked increase in female incidence in 2000 to 5.6 cases per 100 K p-y. Subsequent to 2000, there was a gradual decline in typhoid fever incidence of both genders to less than one case per 100 K p-y by 2002.

**Table 2:** Typhoid cases demographics

Population	S. Typhoid cases	Active duty US military population^a^
<20	1 (0.5)	80,678 (5.7)
20–29	85 (41.5)	784,522 (55.5)
30–39	83 (40.5)	391,404 (27.7)
≥40	36 (17.6)	156,863 (11.1)
Race (*n*, %)		
White	142 (69.3)	983,730 (69.6)
Black	20 (9.8)	234,750 (16.6)
Asian/Pacific Islander	18 (8.8)	66,742 (4.7)
American Indian	1 (0.5)	21,390 (1.5)
Other	19 (9.3)	121,418 (5.5)
Gender (*n*, %)		
Male	176 (85.9)	1,205,034 (85.3)
Female	29 (14.2)	208,465 (14.7)
Education level (v, %)		
High school	105 (51.2)	1,008,843 (71.4)
Some college	36 (17.6)	102,176 (7.2)
Bachelor’s degree	42 (20.5)	169,579 (12.0)
Master’s degree	14 (6.8)	83, 258 (5.9)
Doctorate	2 (1.0)	21,842 (1.6)
Unknown	6 (2.9)	27,801 (2.0)
Rank (*n*, %)		
E1-E4	63 (30.7)	611,370 (43.3)
E5-E9	88 (42.9)	557,516 (39.4)
O1-O5	48 (23.4)	210,124 (14.9)
O6-O10	4 (2.0)	13,433 (1.0)
Warrant officer	2 (1.0)	21,029 (1.5)
Marital status (*n*, %)		
Married	129 (62.9)	791,895 (56.0)
Never married/single	67 (32.7)	620,677 (43.9)
Other	9 (4.4)	927 (0.1)
Branch of service (*n*, %)		
Army	38 (18.5)	533,249 (37.7)
Air force	73 (35.6)	330,214 (23.4)
Marines	14 (6.8)	194,432 (13.8)
Navy	78 (38.1)	314,580 (22.3)
Coast guard	2 (1.0)	41,024 (2.9)

^a^As of April 2013

In evaluating incidence among the different services, the greatest incidence of S. Typhi infection was observed during 1998 in the Air Force with approximately 11.9 cases per 100 K p-y. Second to that, there were approximately nine cases per 100 K p-y observed in the Navy during the year 2000. Following the peak in 2000, annual incidence among all branches was observed to be less than two cases per 100 K p-y. When stratifying incidence by race, Asians had the highest incidence in 2000 with ten cases per 100 K p-y. American Indians had the next highest incidence in 2004, with 5.2 cases per 100 K p-y. Stratifying incidence by age showed the highest incidence among individuals older than 35 in the year 2000. Incidence in the year 2000 among individuals between 35 and 39 was 6.8 cases per 100 K p-y and incidence among individuals aged 40 or greater was 5.2 cases per 100 K p-y.

Of the identified S. Typhi cases, 26 % (*n* = 53) of subjects received a typhoid vaccination of any type within 2 years prior to diagnosis (Table 3). Approximately 63 % (*n* = 130) of subjects had no documentation of S. Typhi vaccination in the 3 years prior to S. Typhi infection and nearly 82 % (*n* = 168) had no documentation of S. Typhi vaccination in the 1 year prior to S. Typhi infection. The most commonly administered vaccine was “any parenteral vaccine other than AKD”, which was administered to 35.3 % (*n* = 72) of subjects. The least commonly administered vaccine was the live oral vaccine, administered to 8.8 % (*n* = 18) of S. Typhi cases. Importantly, the parenteral acetone-inactivated (AKD) vaccine (U.S. armed forces only) and the parenteral H-P vaccine have not been in widespread use since 2005 or earlier [8].

**Table 3:** Number (%) of typhoid fever cases with documented S. Typhi vaccination

Months pre-outcome	Parenteral, other than acetone-killed, dried	Vi capsule	Parenteral acetone-killed dried	Live oral	Unspecified formulation	Any
6	11 (5.4)	17 (8.3)	1 (0.5)	0 (0.0)	0 (0.0)	27 (13.2)
12	16 (7.8)	20 (9.8)	3 (1.5)	0 (0.0)	0 (0.0)	37 (18.1)
18	17 (8.3)	23 (11.3)	4 (2.0)	1 (0.5)	0 (0.0)	43 (21.1)
24	21 (10.3)	25 (12.3)	5 (2.5)	4 (2.0)	0 (0.0)	53 (26.0)
36	32 (15.7)	31 (15.2)	10 (4.9)	5 (2.5)	4 (2.0)	75 (36.8)
48	44 (21.6)	33 (16.2)	15 (7.4)	9 (4.4)	5 (2.5)	94 (46.1)
Any time	72 (35.3)	39 (19.1)	27 (13.2)	18 (8.8)	8 (3.9)	122 (59.8)

Data obtained from CVX codes as follows:

041: typhoid vaccine, parenteral, other than acetone-killed, dried

101: typhoid Vi capsular polysaccharide vaccine

053: typhoid vaccine, parenteral, acetone-killed, dried (U.S. military)

025: typhoid vaccine, live, oral

091: typhoid vaccine, unspecified formulation

## Discussion

We conducted a 13-year retrospective case series study to describe the epidemiological characteristics of S. Typhi infection among active duty military personnel from 1998 to 2011. All subjects were identified as having S. Typhi by ICD9-CM code. We identified a peak incidence of typhoid fever in 2000 followed by a sharp decline in 2001 with incidence remaining below one case per 100 K p-y from 2001 to 2011.

Aside from the rise in incidence in 1998 and 2000, the incidence of typhoid fever in active duty military personnel from 2001 to 2011 was consistent with estimates of incidence in the U.S. during that time period. Approximately 5700 cases of typhoid fever (400 reported) occur annually in the U.S. with the majority of these cases related to travel to endemic areas [1]. Though not directly able to analyze all travel of our population, one hypothesis could be that increased operational deployment of troops to communities endemic with S. Typhi could account for the rise in cases in 1998 and 2011.

There were various deployments of U.S. military personnel to countries with either medium incidence estimates (ten cases per 100 K p-y) or high incidence estimates (100 cases per 100 K p-y) of typhoid fever from 1998 to 2000 such as Kenya, Bosnia, Kosovo, Afghanistan, and Iraq [3, 12]. However, these deployments were relatively small averaging between 2000 and 8000 troops per deployment compared to Operation Enduring Freedom (2001–2014, Afghanistan) and Operation Iraqi Freedom (2003–2010, Iraq), in which over 75,000 troops were deployed in 2003 with the number of deployed personnel increasing to over 180,000 in 2008 and tapering to 100,000 deployed personnel in 2011 [13]. Therefore, given the increase in deployment of troops from 2003 onward, and that travel to regions with higher incidence of typhoid fever is a risk factor for contracting disease, one would anticipate an increased incidence in S. Typhi amongst active duty personnel during those years. One limitation of our data is that deployment data were limited to operational deployments and did not include non-operational deployments or non-deployment related travel. Illustrative of this limitation is that we identified less than 10 S. Typhi cases with a deployment temporally related to diagnosis. Additionally, living conditions for deployed troops may not be comparable to the native population who may lack proper sanitation or access to clean food and water, as there are many measures taken in order to maintain comfort of troops to keep morale high. For example, as early as 2005 there was access to clean food and water in dining facilities, air conditioning, libraries and other recreational facilities on U.S. military bases in Iraq [14]. Certainly, just as travel to areas endemic of typhoid fever is a major risk factor for contracting the disease, we expect that operational deployment to an area endemic of typhoid fever would also increase the risk of disease [4, 5]. However, our data insufficiently characterizes deployment-related exposures as a risk factor for typhoid fever.

Vaccination against S. Typhi provides effective protection for individuals traveling to endemic regions and may account in part for the low incidence of S. Typhi cases seen in years of large operational deployments. For example, in a retrospective study of S. Typhi incidence in vaccinated vs unvaccinated travelers in Nepal, 88–100 % of travelers from North America, Western Europe and Australia were vaccinated, and vaccines were estimated to be 95 % effective [15]. In contrast, of the 205 active duty servicemen found to have an S. Typhi associated illness, only 26.0 % (*n* = 53) had a documented S. Typhi vaccination within 2 years of diagnosis.

Interestingly, only 18.1 % (*n* = 37) of subjects received a typhoid vaccine within 1 year of diagnosis of S. Typhi. Though not able to directly estimate vaccine efficacy, the low prevalence of Typhoid in recently vaccinated subjects may reflect the relatively high efficacy of these vaccines, or alternatively, the broad-based coverage of S. typhi vaccines in this population.

The most commonly administered vaccine was “any parenteral vaccine other than AKD”, which was administered to 35.3 % (*n* = 72) of subjects. However, the data of vaccines was categorical and linked to vaccination codes, and therefore information is limited on what specific vaccinations are included in the category “any parenteral vaccine other than AKD.” This category may include both the Vi polysacharride vaccine and the heat-phenol (H-P) vaccine, or may just be the H-P vaccine alone. Nevertheless, 3-year cumulative efficacy rates for the Vi vaccine and the H-P vaccine are estimated to be 30–70 % and 51–77 %, respectively [7, 10]. Although our data do not necessarily demonstrate the protective effect of typhoid vaccine, the current military policy of vaccinating all active duty servicemen before deployment to areas endemic of S. Typhi likely lowers the incidence of S. Typhi amongst this population, given the high efficacy of existing typhoid vaccines.

Clinically, patients infected with S. Typhi or S. Paratyphi present with nearly identical symptoms and are categorized as having typhoid or paratyphoid fever based on diagnostic testing results [6, 16, 17]. Diagnosis can be made by isolating the organism from blood, stool or bone marrow or through serological testing, however these tests vary in reliability and in the manner in which they are gathered [2, 18]. When culturing for S.Typhi or S. Paratyphi, the sensitivity of blood culture ranges between 20 and 66 % and the sensitivity of bone marrow culture ranges from 48 to 100 % [6, 18–20]. Additionally, logistical constraints limit the availability of culture capability in deployed settings increasing the difficulty of S. Typhi diagnosis [6]. New, rapid, kit-based serologic tests with higher sensitivity may improve diagnostic capabilities in specific scenarios [2]. Our cases were identified by ICD9-CM code, which may or may not have included the aforementioned confirmatory testing and does not include information on location of diagnosis. Therefore, the sensitivity and specificity of diagnostic testing used to confirm S. Typhi infection may have varied from patient to patient and it is possible that subjects identified as having S. Typhi, may actually have been infected with S. Paratyphi or have been false positives. Distinguishing S. Paratyphi infection from S. Typhi may help to better characterize the burden and aetiology of enteric fever amongst active duty military personell [3, 21, 22].

## Conclusion

Although limitations of this retrospective study include the utilization of medical encounter data obtained from ambulatory and inpatient claims data, limited information about method of diagnosis, vaccine status, deployment locations, and possible selection bias, this series of cases reveals important epidemiological information that may help guide preventive care against S. Typhi infection amongst active duty servicemen. In order to best guide preventive care, further research into deployment history, vaccine utilization, and incidence of S. Paratyphi infection should be explored.
